# Impact Factors and an Efficient Nomogram for Predicting the Occurrence of Sepsis after Percutaneous Nephrolithotomy

**DOI:** 10.1155/2020/6081768

**Published:** 2020-12-22

**Authors:** Jun Wang, Yuanyuan Mi, Sheng Wu, Hongbao Shao, Lijie Zhu, Feng Dai

**Affiliations:** Department of Urology, Affiliated Hospital of Jiangnan University, Wuxi 214100, China

## Abstract

**Introduction:**

To describe the clinical parameters of urinary stones and investigate the preoperative predictors of sepsis in patients following percutaneous nephrolithotomy (PCNL). *Patients and Methods*. A retrospective study of patients who underwent PCNL between August 2017 and December 2019 was performed. The patients were divided into the sepsis and nonsepsis groups according to whether they had sepsis, and their data were compared for further analysis.

**Results:**

Fifteen (6.1%) patients matching in age, gender, body mass index (BMI), and the number of access variables were included in the sepsis group. The multivariate analysis demonstrated that the staghorn calculi (OR: 12.206, *P* < 0.001) and positive midstream urine culture (OR: 16.505, *P* < 0.001) were independent risk factors of sepsis, while preoperative renal fistula (OR: 0.122, *P* < 0.001) was a protective factor of sepsis. The three factors were ultimately selected to develop a nomogram to predict the probability of sepsis. The new nomogram was well calibrated and had higher diagnostic accuracy (the area under the curve: 0.916).

**Conclusions:**

Our study reveals that patients with complex stones and positive bacteriuria are associated with a significantly high risk of sepsis after surgery. The removal of obstruction before operation under certain conditions might be a reliable protective factor of sepsis.

## 1. Introduction

Urinary stones are one of the most common diseases, with an incidence of approximately 5-15% around the world [1, 2] and a recurrence rate reaching 50% in a decade [3, 4]. Due to the advantages of high effectiveness in stone removal and quick recovery, percutaneous nephrolithotomy (PCNL) is now recognized as the first-line choice for the removal of renal stones, especially for complex stones with a high burden [5–7]. Nevertheless, PCNL can also induce severe adverse events occasionally.

Sepsis, a systemic inflammatory response syndrome (SIRS) clinically confirmed or highly suspicious by documented evidence of bacterial pathogens, is a notable postoperative complication after PCNL with an incidence of about 0.3% to 7.6% [8]. Based on the Sequential Organ Failure Assessment (SOFA) scoring system, sepsis is defined as life-threatening organ dysfunction caused by a dysregulated host response to infection [9]. It is the most common cause of perioperative death in PCNL in large series [7]. Some important risk factors of infection after PCNL, including the stone size and operation time, have been well identified [8]. However, the evaluation of effect factors for sepsis or a few risk factors that only apply to struvite patients undergoing PCNL is rarely studied [10]. Therefore, in the present study, the related factors for urinary sepsis were evaluated, and a new nomogram was developed to predict the probability of sepsis for all post-PCNL patients.

## 2. Methods

### 2.1. Study Design and Data Collection

This paper retrospectively reviewed the data of consecutive patients with the renal calculi or upper urethral calculi who underwent PCNL at the Affiliated Hospital of Jiangnan University from August 2017 to December 2019. All operations were performed in standard procedure by experienced urologists. Patients who had tumor, blood diseases, urinary tract tuberculosis, and juvenile and incomplete medical records were excluded. Patients' preoperative factors including age, sex, BMI, diabetes, surgery time, serum creatinine (SCr), midstream urine culture, stone location, the urinary sediment microscopy WBC (+ ∼ ++++), and staghorn calculi were recorded. Appropriate antibiotics would be administered as empirical therapy when patients had any focus of infection, and the use of sensitive antibiotics was guided by the positive urine culture report before operation. Sepsis was defined by a SOFA score of 2 or more consequent upon confirmed or suspected infection [9]. (Supplementary Table 1).

### 2.2. Percutaneous Nephrolithotomy Technique

After induction of general anesthesia in the lithotomic position, a urethral catheter was placed, and the patient was moved to a prone position for the acquisition of percutaneous access under ultrasonographic guidance. Then, 22F–24F Amplatz working sheaths were inserted with the help of a guide wire through the track enlarged by balloon dilatation. After performing 17F nephros-copy and 1000 *μ*m holmium laser fiber, or 22F nephroscopy and ultrasonic endoscopic lithotripter to maximize the dusting effect, the stone fragments were flushed out by irrigation, forceps, or graspers. At the end of the procedure, a 5F double-J stent and an 18F–20F nephrostomy tube were placed for drainage, which usually were left in the body for 2-4 weeks and 7 days, respectively.

### 2.3. Statistical Analysis

SPSS20.0 (SPSS, Chicago, IL, USA) was used for statistical analysis. Data were collected as mean plus or minus the standard deviation (SD) or the median and range. Comparisons of normally distributed continuous variables were performed using Student's *t*-test. Categorical variables were presented as numbers (percentages) and analyzed using the chi-square test or Fisher's exact test. The relative risk (odds ratio, OR) was used in a multivariable logistic regression by backward stepwise to determine risk factors for sepsis. Two-tailed *P* values <0.05 were considered statistically significant. A nomogram for sepsis was established based on the multivariate analysis results achieved by *R* software (Version 3.4.1; Institute for Statistics and Mathematics, Vienna, VIC, Austria) with the package rms.

## 3. Results

A total of 246 patients (184 males, 62 females) successfully treated with minimally invasive percutaneous nephrolithotomy were included in this retrospective study, with an average age of 51.22 ± 10.48 (range: 25 to 77 years). Fifty-five patients (22.4%) had diabetes mellitus. There were 127 right kidney stones, 84 left kidney stones and 35 bilateral kidney stones. Twenty-one cases had staghorn stones. The types of stones included renal pelvis stones (75 cases, 30.5%), calyceal stones (104 cases, 42.3%), and upper ureteral stones (67 cases, 27.2%). The average operation time was 87.93 ± 32.95 minutes (range: 30 to 190 minutes). The preoperative positive midstream urine culture was noted in 18 (7.32%) patients, the urinary sediment microscopy WBC (+ ∼ ++++) was observed in 201 (81.71%) patients, and the preoperative renal fistula (nephrostomy) was calculated in 10 (4.07%) patients (Table 1). In the present study, 15 of 246 patients (6.1%) were found to be sepsis based on the sepsis-3 definition [9].

The patients were divided into the sepsis group and the nonsepsis group according to their complications after surgery. The mean ages of the sepsis and nonsepsis groups were 49.5 ± 10.20 and 52.47 ± 10.52 years, respectively, which showed no significant difference (*P* = 0.322). Besides, no statistically significant difference was found between the two groups in gender, stone location, BMI, the stone side, the stone number, serum creatinine, and surgery time (all *P* > 0.05). However, staghorn calculi, a larger stone size, positive midstream urine culture, diabetes mellitus, the urinary microscopy WBC (3+), and preoperative renal fistula were more frequently observed in patients with sepsis than in those without sepsis after surgery (all *P* < 0.05) (Table 2).

In the univariate analysis, a significant correlation was observed between sepsis and six variables, which were stone location (*P* = 0.048), stone side (*P* = 0.039), staghorn calculi (*P* < 0.001), midstream urine culture (*P* < 0.001), a large stone size (*P* = 0.029), and preoperative renal fistula (*P* < 0.001). There was no statistically significant difference between the two groups in age, gender, BMI, diabetes mellitus, serum creatinine, the stone number, surgery time, and the urinary microscopy WBC (*P* > 0.05).

In the multivariate logistic regression analysis, staghorn calculi [OR = 12.206, 95%confidence interval (CI) = 3.866 − 38.536, *P* < 0.001], positive midstream urine culture (OR = 16.505, 95%CI = 5.582 − 48.798, *P* < 0.001), and preoperative renal fistula (OR = 0.122, 95%CI = 0.032 − 0.463, *P* < 0.001) were identified as either independent risk or protective factors of postoperative sepsis (Table 3).

Based on the above results, the predictive equation can be described as risk index = −1.148 + 2.757 × (staghorn calculi) + 2.636 × (midstream urine culture) − 2.649 × (preoperative renal fistula).

**Figure 1:**
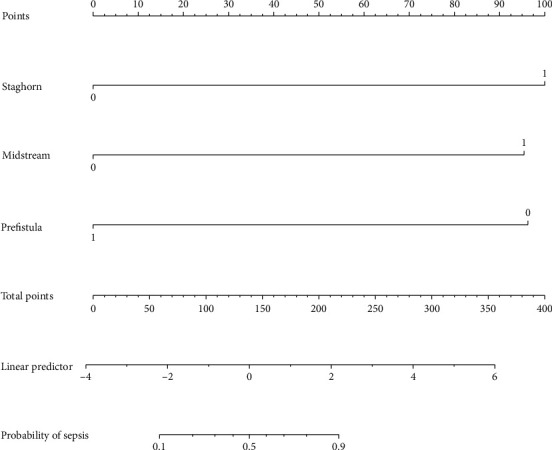
Nomogram with staghorn calculi, positive preoperative midstream urine culture, and preoperative renal fistula predicts the probability of sepsis.

**Figure 2:**
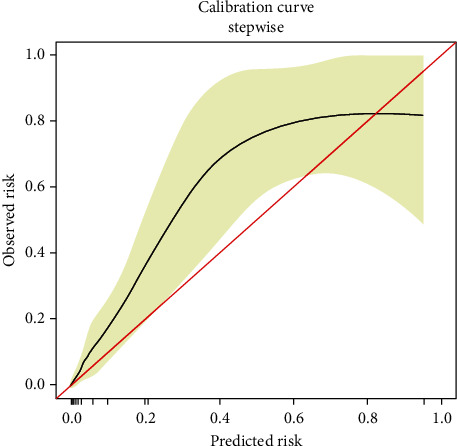
Calibration plot of the nomogram for the probability of sepsis.

**Figure 3:**
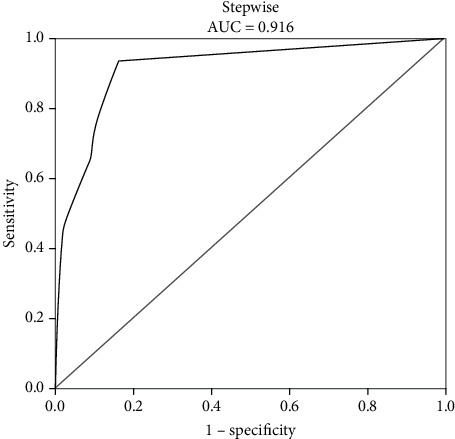
ROC of our new model.

The nomogram was depicted by three independent predictors described in the multivariate analysis (Figure 1). The sum of points in the nomogram demonstrated the probability of sepsis (the bottom scale). The nomogram was internally validated by computing the bootstrap-corrected Harrell index and by the calibration plot. As illustrated in Figure 2, the model had moderate discriminative ability and was well calibrated. The area under the receiver-operating characteristic curve (AUC) of the new nomogram was 0.916 (Figure 3).

## 4. Discussion

Percutaneous nephrolithotomy is a minimally invasive and effective treatment for kidney stones and usually the preferred therapy for complex upper urinary tract calculi [11]. However, this gold standard treatment still has the potential risk of causing complications, of which the incidence reaches 8-38% [12]. The stone-colonizing bacteria released during PCNL translocate through systemic circulation, contributing to the occurrence of postoperative infections and even sepsis [13]. In our research, the incidence of postoperative sepsis was 6.09%, and its outcome was similar to that found by previous studies [8].

In this retrospective study, the multivariate logistic regression analysis of patients' clinical data collected showed that staghorn calculi, preoperative positive midstream urine culture, and preoperative renal fistula were independently related to postoperative sepsis, whereas no significant association of sepsis with serum albumin and the urinary sediment microscopy WBC was observed. This result conflicted with that achieved by prior studies [10, 14].

Aimed at removing stones, relieving obstruction, and protecting renal function, the PCNL surgery needed to reach the renal pelvic, renal calyx, or upper urinary tract, possibly resulting in an increased chance of infections [15, 16]. Compared with small calculi, the large calculi caused relatively prolonged operation time and increased incidence of sepsis, as confirmed by our single factor analysis, but the results of the multivariate logistic regression analysis indicated that the stone location and stone size were not independent risk factors. This result was consistent with that obtained by the study of Lojanapiwat et al. [17].

Staghorn calculi are defined as the stones with multiple branches occupying a part or all of the renal pelvis and renal calyces [18]. Recently, Rivera et al. have retrospectively investigated 277 patients who underwent PCNL and identified 37 (16%) patients with infectious complications. Their results demonstrated that the presence of a staghorn calculus remained independently associated with the increased risk of fever/SIRS/sepsis (OR: 3.14) [13]. Due to differences in the diagnostic criteria of sepsis and heterogeneity of the study population, some letters did not support the above conclusion [10, 19] .Our multivariate analysis suggested that the staghorn calculi probably evolved into sepsis (OR: 12.206).

The preoperative midstream urine culture and intraoperative renal pelvic urine culture have been indicated as significant risk factors for post-PCNL sepsis [20]. Consistent with the result of previous studies, the positive urine culture was found to be associated with urosepsis and identified as an independent risk factor in the multivariate analysis of this study (OR: 16.505). Therefore, to avoid sepsis after PCNL patients with positive preoperative bacteriuria must be accurately treated by antibiotics sensitive for the detected pathogens before the surgical treatment.

Preoperative infections can be caused by kidney swelling (hydronephrosis) that may be associated with high renal pelvis pressure, especially in patients with serious hydronephrosis or basic kidney diseases. These patients are often suggested to remove obstruction before surgery, which may reduce the incidence of postoperative infections. As it was speculated, our results revealed that the preoperative renal fistula remained independently associated with sepsis. In addition, it was worth noting that preoperative renal fistula would be a protective factor of sepsis (OR: 0.122), which may be attributed to the removal of the bacteria culture medium. The protective role of preoperative renal fistula has not been reported in previous studies [10, 14], but it only applies to specific patients.

To the best of our knowledge, nomograms have been widely used in clinical studies [21, 22]. A recent study by Gao et al. [10] has investigated the predictors of sepsis among patients with struvite stones and performed the nomogram based on the calculated scores of some related factors (preoperative serum creatinine and multidrug resistance) to predict the probability of sepsis. However, this model included limited factors and was only for patients with struvite stones. Based on this model, we established a meaningful nomogram that contained two risk factors (staghorn calculi and preoperative positive midstream urine culture) and a protective factor (preoperative renal fistula) for the prediction of the probability of sepsis, and the area under the receiver-operating characteristic curve of the nomogram was calculated, which was 0.916, indicating high diagnostic accuracy.

This study has some distinct limitations. It was a retrospective design with a single institution. Besides, the sample size was small, which might be ascribed to the decreased number of more invasive PCNL surgery due to the more frequent application of flexible ureteroscope lithotripsy. What is more, our new nomogram was based on several ready predictors because some novel gene biomarkers and factors such as CaSR986 gene polymorphism and C-reactive proteins [23, 24] were not included in this study due to health care restrictions in China.

## 5. Conclusion

In summary, the staghorn calculi and positive preoperative midstream urine culture are two risk factors of sepsis after percutaneous nephrolithotomy, and preoperative renal fistula is a protective factor of sepsis. The results may provide new insights for us into the prevention of postoperative complications. Furthermore, the nomogram developed in the current study is a moderately accurate tool that can predict the probability of sepsis for patients after PCNL surgery.

## Figures and Tables

**Table 1:** Basic clinical parameters into the group of patients.

Parameter	Mean ± SD or *n* (%)	Median (range)
Male : female (*n*)	184 (74.8)/62 (25.2)	
Age (years)	51.22 ± 10.48	52 (25-77)
Diabetes mellitus (*n*)	55 (22.4)/191 (77.6)	
Stone location		
Renal pelvis	75 (30.5)	
Calyceal	104 (42.3)	
Upper ureter	67 (27.2)	
BMI(kg/m^2^)	24.09 ± 2.80	24.22 (17.36-31.22)
Right/left/two sides (*n*)	127 (51.6)/84 (34.1)/35 (14.3)	
Stone number, single/multiple	131 (53.3)/115 (46.7)	
Staghorn calculi (*n*)	36 (17.1)/210 (82.9)	
Largest stone size (mm)	24.22 ± 9.91	20.5(8-67)
Serum creatinine (umol/L)	80.33 ± 35.17	72.75 (42.5-329)
Midstream urine culture		
Positive	18 (7.3)	
Negative	228 (92.7)	
The urinary microscopy WBC		
WBC (-)	45 (18.3)	
WBC (+)	32 (13.0)	
WBC (++)	51 (20.7)	
WBC (+++)	19 (7.8)	
WBC (++++)	99 (40.2)	
Preoperative renal fistula, yes/no	10 (4.1)/236 (95.9)	
Surgery time (min)	87.93 ± 32.95	82.5 (30-190)

BMI: body mass index. The urinary microscopy WBC : high magnification, urine sediment white blood cell count. WBC (+): urinary sediment microscopy counts of 5—10/HP. WBC (++): urinary sediment microscopic counting 10—15/HP. WBC (+++): urinary sediment microscopic counting 15—20/HP. WBC (++++): urinary sediment microscopic counting >20/HP.

**Table 2:** Clinical and preoperative factors between sepsis and nonsepsis groups.

Variables	Sepsis	Nonsepsis	*t*/*χ*2	*P* value
Age (years)	49.5 ± 10.20	51.47 ± 10.52	-0.993	0.322
Gender			1.855	0.173
Male	9 (60.0)	175 (73.5)		
Female	6 (40.0)	56 (26.5)		
Diabetes mellitus			5.438	0.028^∗^
Yes	7 (46.7)	48 (20.8)		
No	8 (53.3)	183 (79.2)		
Stone location			0.867	0.648
Renal pelvis	3 (20.0)	72 (31.2)		
Calyceal	7 (46.7)	97 (42.0)		
Upper ureter	5 (33.3)	62 (26.8)		
BMI(kg/m^2^)	24.48 ± 2.74	24.03 ± 2.81	0.850	0.396
Stone side			0.953	0.621
Right	6 (40.0)	121(52.4)		
Left	6 (40.0)	78 (33.8)		
Two sides	3 (20.0)	32 (13.8)		
Stone number			1.155	0.283
Single	10 (66.7)	121 (52.4)		
Multiple	5 (33.3)	110 (47.6)		
Staghorn calculi			85.899	<0.001^∗^
Yes	11 (73.3)	10 (4.3)		
No	4 (26.7)	221 (95.7)		
Largest stone size (mm)	27.84 ± 14.77	23.63 ± 8.85	2.265	0.024^∗^
Serum creatinine (umol/L)	80.29 ± 24.72	80.33 ± 36.52	-0.007	0.995
Midstream urine culture			82.968	<0.001^∗^
Positive	10 (66.7)	8(3.50)		
Negative	5 (33.3)	223(96.5)		
The urinary microscopy WBC			11.005	0.027^∗^
WBC (+)	1 (6.6)	31 (13.4)	1.425	0.233
WBC (++)	3 (20.0)	48 (20.8)	2.732	0.098
WBC (+++)	4 (26.7)	15 (6.50)	10.105	0.001^∗^
WBC (++++)	7 (46.7)	92 (39.8)	3.344	0.067
Preoperative renal fistula			52.894	<0.001^∗^
Yes	6 (40.0)	4 (1.8)		
No	9 (60.0)	227 (98.2)		
Surgery time (min)	88.50 ± 30.28	87.85 ± 33.40	0.104	0.977

^∗^Statistically significant, BMI: body mass index. The urinary microscopy WBC: at high magnification, urine sediment white blood cell count. WBC (+): urinary sediment microscopy counts of 5—10/HP. WBC (++): urinary sediment microscopic counting 10—15/HP. WBC (+++): urinary sediment microscopic counting 15—20/HP. WBC (++++): urinary sediment microscopic counting >20/HP.

**Table 3:** Univariate analysis and multivariate binary logistic regression analysis.

Variable	Univariate analysis			Multivariate analysis		
	OR	95% CI	*P*	OR	95% CI	*P*
Age(years)	0.982	0.948-1.018	0.321			
BMI	1.059	0.927-1.210	0.395			
Gender	1.674	0.756-3.705	0.204			
Male/female						
Diabetes mellitus	0.844	0.356-2.001	0.701			
Yes/no						
Stone location	1.667	1.005-2.763	0.048^∗^			
Renal pelvis/calyceal/upper ureter						
Stone side	1.675	1.027-2.734	0.039^∗^			
Left/right/two sides						
Stone number	1.006	0.478-2.118	0.998			
Single/multiple						
Staghorn calculi	8.964	4.011-20.032	<0.001^∗^	10.402	3.663-29.542	<0.001^∗^
Yes/no						
Largest stone size	1.036	1.004-1.070	0.029^∗^			
Serum creatinine	1.000	0.989-1.011	0.995			
Midstream urine culture	15.911	6.703-37.768	<0.001^∗^	16.505	5.582-48.798	<0.001^∗^
Positive/negative						
Urinary microscopy WBC	0.506	0.159-1.611	0.747			
1+/2+/3+/4+						
Preoperative renal fistula	0.055	0.018-0.164	<0.001^∗^	0.122	0.032-0.463	0.002^∗^
Yes/no						
Surgery time	1.001	0.989-1.012	0.917			

^∗^Statistically significant, BMI: body mass index. The urinary microscopy WBC: at high magnification, urine sediment white blood cell count. WBC (1+): urinary sediment microscopy counts of 5—10/HP. WBC (2+): urinary sediment microscopic counting 10—15/HP. WBC (3+): urinary sediment microscopic counting 15—20/HP. WBC (4+): urinary sediment microscopic counting >20/HP.

## Data Availability

The data are available on request.

## Supplementary Materials

Supplementary Materials Supplementary Table 1: components of the SOFA score.
