# Supplementary material for: Impact Factors and an Efficient Nomogram for Predicting the Occurrence of Sepsis after Percutaneous Nephrolithotomy
Source: Biomed Res Int. 2020 Dec 22;2020:6081768. doi: 10.1155/2020/6081768 (PMC7772030; doi:10.1155/2020/6081768)
Supplement: Supplementary Materials — Supplementary Table 1: components of the SOFA score. [file 6081768.f1.docx]

**Supplementary Table 1. Components of SOFA Score**

| Parameters and score | Parameters and score |
| --- | --- |
| SOFA Score | Respiration, PaO2/FIO2, mmHg |
| 0 | 0 (≥400) |
| 1 | 1 (<400) |
| 2 | 2 (<300) |
| 3 | 3(<200 with respiratory support) |
| 4 | 4(<100 with respiratory support) |
| ≥5 |  |
| Coagulation, platelets,×10^3^/μL | Liver, bilirubin, mg/dL |
| 0 (≥150) | 0 (1.2) |
| 1 (<150) | 1 (1.2–1.9) |
| 2 (<100) | 2 (2.0–5.9) |
| 3 (<50) | 3 (6.0–11.9) |
| 4 (<20) | 4 (>12.0) |
| Cardiovascular | Glasgow Coma Scale Score ^b^ |
| 0 (MAP≥70 mmHg) | 0 (15) |
| 1 (MAP<70 mmHg)  2 (Dopamine<5 or dobutamine (any dose))^a^ | 1 (13–14)  2 (10–12) |
| 3 (Dopamine 5.1–15 or epinephrine≤0.1 or norepinephrine≤0.1)^a^  4 (Dopamine>15 or epinephrine>0.1 or norepinephrine>0.1)^a^ | 3 (6–9)  4 (<6) |
| Renal, creatinine, mg/dL (urine output, ml/d) |  |
| 0 (<1.2) |  |
| 1 (1.2–1.9) |  |
| 2 (2.0–3.4) |  |
| 3 (3.5–4.9) (<500) |  |
| 4 (>5) (<200) |  |

FIO2 fraction of inspired oxygen, MAP mean arterial pressure, PaO2 partial pressure of oxygen
^a^Catecholamine doses are given as μg/kg/min for at least 1 h

^b^Glasgow Coma Scale Scores range from 3 to 15; higher score indicates better neurological function
